# Severe intestinal dysbiosis is prevalent in primary Sjögren’s syndrome and is associated with systemic disease activity

**DOI:** 10.1186/s13075-017-1446-2

**Published:** 2017-10-24

**Authors:** Thomas Mandl, Jan Marsal, Peter Olsson, Bodil Ohlsson, Kristofer Andréasson

**Affiliations:** 1 Section of Rheumatology, Department of Clinical Sciences Malmö, Lund University, Malmö, Sweden; 2 Department of Gastroenterology, Skane University Hospital, Lund, Sweden; 3 Immunology Section, Department of Experimental Medical Science, Lund University, Lund, Sweden; 4 Section of Medicine, Department of Clinical Sciences Lund, Lund University, Lund, Sweden; 5 Section of Internal Medicine, Department of Clinical Sciences Malmö, Lund University, Malmö, Sweden; 6 Section of Rheumatology, Department of Clinical Sciences Lund, Lund University, Lund, Sweden; 7 Department of Rheumatology, Skane University Hospital Malmö, Inga Marie Nilssons gata 32, S-205 02 Malmö, Sweden

**Keywords:** Primary Sjögren’s syndrome, Microbiome, Gastrointestinal, Dysbiosis, Disease activity

## Abstract

**Background:**

Altered microbial composition of the intestine, commonly referred to as dysbiosis, has been associated with several autoimmune diseases including primary Sjögren’s syndrome (pSS). The aims of the current study were to study the intestinal microbial balance in pSS and to identify clinical features associated with dysbiosis.

**Methods:**

Forty-two consecutive pSS patients and 35 age-matched and sex-matched control subjects were included in the study in an open clinic setting. Stool samples were analyzed for intestinal dysbiosis using a validated 16S rRNA-based microbiota test (GA-map™ Dysbiosis Test; Genetic Analysis, Oslo, Norway). Dysbiosis and severe dysbiosis were defined in accordance with the manufacturer’s instructions. Patients were evaluated with regard to disease activity (European League Against Rheumatism (EULAR) Sjögren’s Syndrome Disease Activity Index (ESSDAI) and Clinical ESSDAI (ClinESSDAI)). In addition, patients were examined for laboratory and serological features of pSS as well as fecal calprotectin levels.

Furthermore, patients were investigated regarding patient-reported outcomes for pSS (EULAR Sjögren’s Syndrome Patient Reported Index (ESSPRI)) and irritable bowel syndrome (IBS)-like symptoms according to the Rome III criteria.

**Results:**

Severe dysbiosis was more prevalent in pSS patients in comparison to controls (21 vs 3%; *p* = 0.018). Subjects with pSS and severe dysbiosis had higher disease activity as evaluated by the ESSDAI total score (13 vs 5; *p* = 0.049) and the ClinESSDAI total score (12 vs 5; *p* = 0.049), lower levels of complement component 4 (0.11 vs 0.17 g/L; *p* = 0.004), as well as higher levels of fecal calprotectin (110 vs 33 μg/g; *p* = 0.001) compared to the other pSS patients. In contrast, severe dysbiosis among pSS patients was not associated with disease duration, IBS-like symptoms, or the ESSPRI total score.

**Conclusions:**

Severe intestinal dysbiosis is a prevalent finding in pSS and is associated both with clinical and laboratory markers of systemic disease activity as well as gastrointestinal inflammation. Further studies are warranted to elucidate a potential causative link between dysbiosis and pSS.

## Background

Primary Sjögren’s syndrome (pSS) is a systemic autoimmune disease, characterized by lymphocytic infiltration of exocrine glands resulting in exocrine hypofunction and sicca symptoms. The disease may also result in various extraglandular manifestations (EGM) such as arthritis, pulmonary involvement, renal disease, vasculitis, and neuropathy [1, 2]. The gastrointestinal (GI) tract may also be involved and esophageal dysmotility [3, 4], gastroparesis [5], atrophic gastritis [6], and pancreatic insufficiency [6] are commonly encountered in pSS.

Increased levels of fecal calprotectin (F-calprotectin), a validated marker for GI inflammation, have been found in a subgroup of pSS patients and were associated with concomitant organic GI disease [7]. GI complaints are common in pSS and a large proportion of pSS patients exhibit symptoms typically observed in irritable bowel syndrome (IBS) or dysmotility [5, 8, 9].

Recently, increasing interest has been directed against the importance of the GI microbiota and its influence on autoimmune disease [10]. In both health and disease, there is a continuous interaction between the microbiota, the epithelium, and the immune cells of the GI mucosa. This interaction has been suggested to have profound effects on both the local and systemic immune system [11], and may both curtail and amplify local and systemic inflammatory disease [12]. Because the GI epithelium is affected in pSS, through the inflammation of the exocrine glands as well as through diminished secretions, there are decreased levels of both protective and proliferative factors [6, 13, 14]. Consequently, the integrity of the epithelium and its barrier function may be compromised in pSS patients, possibly resulting in a perturbed microbiota–host immune system interaction.

An altered microbiota composition, commonly referred to as dysbiosis, has been shown to induce and modulate systemic inflammation in experimental animal models of rheumatic diseases [15], including pSS [16]. In the clinical setting, intestinal dysbiosis has been associated with several autoimmune diseases including rheumatoid arthritis, systemic lupus erythematosus, ankylosing spondylitis, celiac disease, autoimmune hepatitis, systemic sclerosis, and pSS [10, 15, 17–21]. Recently, de Paiva et al. demonstrated that intestinal dysbiosis may worsen experimental pSS in mice. They also reported an association between pSS and intestinal dysbiosis in a small number of patients (*n* = 10) [16].

The aims of this study were to explore intestinal microbial balance in pSS and to relate these findings to clinical features of disease.

## Methods

### Patients

Forty-two consecutive outpatients (median age 62, range 24–80 years; 40 females) diagnosed with pSS according to the American–European Consensus Group (AECG) criteria [22], but also fulfilling the American Congress of Rheumatology–European League Against Rheumatism (EULAR) criteria for pSS [23], seen at the Department of Rheumatology, Skane University Hospital, Malmö, Sweden, were included in the study. Concurrent inflammatory bowel disease (IBD) and antibiotic treatment during the last 3 months served as exclusion criteria. Patients’ characteristics are presented in Table 1.

**Table 1:** Patient characteristics

	pSS patients (*N* = 42)
Age (years)	62 (51; 68)
Sex (males/females)	2/40
Disease duration (years)	16 (7; 23)
Current/prior/never smokers (%)	20/41/39
Anti-SS-A antibody seropositives (%)	76
Anti-SS-B antibody seropositives (%)	48
ANA seropositives (%)	79
RF seropositives (%)	52
IgG (g/L)	13.1 (10.0; 17.1)
Complement component 3 (g/L)	1.00 (0.89; 1.15)
Complement component 4 (g/L)	0.16 (0.12; 0.21)
Lip biopsy—focus score ≥ 1 (*N* positives/*N* studied (%))	28/33 (85)
ESSDAI total score	6 (1; 12)
ESSPRI total score	6 (5; 8)
Fecal calprotectin (μg/g)	38 (20; 123)
Irritable bowel syndrome-like symptoms (%)	42
Using proton pump inhibitors (%)	36
Using NSAIDs (%)	38
Using glucocorticoids (%)	36
> 5 mg prednisolone daily	7
5 mg prednisolone daily	24
< 5 mg prednisolone daily	5
Using anti-malarials (%)	29
Using other DMARDs (%)	2

Demographic and clinical characteristics of the patients with primary Sjögren’s syndrome (*pSS*). Results presented as median (interquartile range) or percentage of subjects

*DMARD* disease-modifying anti-rheumatic drug, *ESSDAI* EULAR Sjögren’s Syndrome Disease Activity Index, *ESSPRI* EULAR Sjögren’s Syndrome Patient Reported Index, *EULAR* European League Against Rheumatism, *NSAID* nonsteroidal anti-inflammatory drug, *ANA* antinuclear antibodies, *RF* rheumatoid factor

### Control group

Thirty-five age-matched and sex-matched control subjects (median age 62, range 39–78 years; 33 females) consisting of hospital workers, their relatives, and their friends were invited and accepted for this study. Subjects with any rheumatologic or IBD diagnosis, as well as concurrent antibiotic treatment, were excluded.

### Clinical assessment

The patients were evaluated for systemic disease manifestations by the EULAR Sjögren’s Syndrome Disease Activity Index (ESSDAI) [1] and the Clinical ESSDAI (ClinESSDAI) [24]. Patient-reported outcomes, including symptoms of sicca, pain, and fatigue, were evaluated by the EULAR Sjögren’s Syndrome Patient Reported Index (ESSPRI) [25]. In addition, the presence of IBS-like symptoms, as defined by the Rome III criteria, was evaluated by a validated questionnaire on GI complaints [26]. Concomitant use of proton pump inhibitors (PPI), nonsteroidal anti-inflammatory drugs (NSAIDs), glucocorticoids (GCs), anti-malarials (AMA), and other disease-modifying anti-rheumatic drugs (DMARDs) was noted.

### Laboratory analyses

Laboratory testing included measurement of levels of IgG, as well as analysis of autoantibodies including anti-SS-A and anti-SS-B antibodies, ANA, and RF. Complement levels were assessed by measurement of complement component 3 (C3) and complement component 4 (C4). F-calprotectin, a marker of GI inflammation, was measured with a commercially available enzyme-linked immunosorbent assay using a monoclonal antibody (Bühlmann Laboratories, Schönenbuch, Switzerland). All analyses were performed at the Department of Laboratory Medicine, Skane University Hospital.

### Evaluation of intestinal dysbiosis

The GA-map™ Dysbiosis Test (Genetic Analysis, Oslo, Norway) has been developed in order to identify and grade intestinal dysbiosis by analysis of microbial genes in a stool sample. The test makes use of 54 bacterial 16S rRNA probes specific for various intestinal bacterial species or clades to generate data on the intestinal microbiota composition. Using a defined algorithm, these data are subsequently translated into a Dysbiosis Index Score (DIS) ranging from 1 to 5. The test has been compared with MiSeq Illumina sequencing-based protocols and proven successful in identifying dysbiosis [21, 27–29]. In a previous study of a population consisting of young healthy adults, 84% exhibited DIS 1–2 and 16% exhibited DIS ≥ 3 [27]. In the current study, dysbiosis was defined as DIS ≥ 3 and severe dysbiosis as DIS 5, in accordance with the manufacturer’s instructions.

### Ethics

The study was approved by the Regional Ethics Review Board, Lund, Sweden (LU 2011/596). All patients and controls gave written informed consent and the study was performed in accordance with the declaration of Helsinki.

### Statistical analyses

The Mann–Whitney *U* test was used when comparing continuous variables and the chi-square test or Fisher’s exact test for comparing discrete variables and frequency distribution analysis. Correlations were evaluated by Spearman rank correlation. Values are presented as median and interquartile range (IQR) or number and percentage of subjects. *p* < 0.05 was considered statistically significant.

## Results

**Fig. 1:**
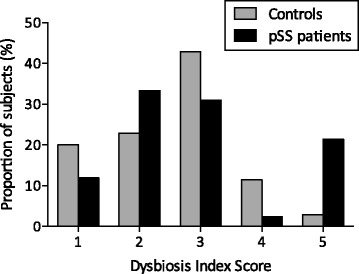
Dysbiosis Index Score in primary Sjögren’s syndrome and age-matched and sex-matched control subjects. A subgroup of patients with severe dysbiosis (DIS 5) can be identified among the subjects with primary Sjögren’s syndrome (pSS) but not in the control group

Eubiosis and moderate dysbiosis were common in both the pSS group and the control group. However, the frequency distribution among the pSS patients was different from that of the controls (*p* = 0.045) (Fig. 1). A large subgroup of pSS patients exhibited severe dysbiosis (DIS 5) which was not the case among controls (21 vs 3%; *p* = 0.018) (Fig. 1). Consequently, we chose to analyze this pSS subgroup in relation to the other pSS subjects.

**Fig. 2:**
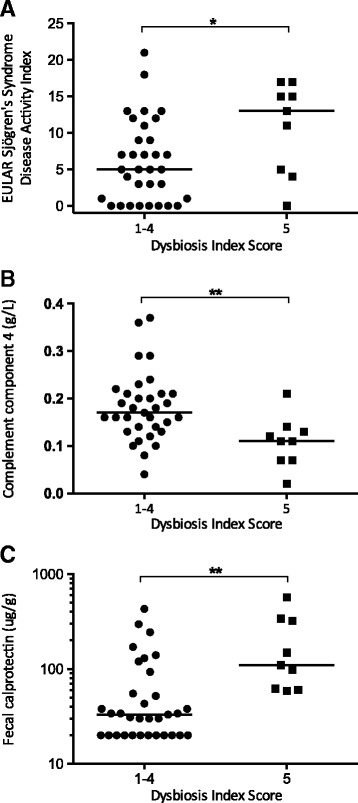
Disease activity (**a**), hypocomplementemia (**b**), and intestinal inflammation (**c**) in pSS subjects with and without severe dysbiosis, defined as a Dysbiosis Index Score 5. Patients with intestinal dysbiosis have higher ESSDAI scores (**a**), lower C4 levels (**b**), and higher levels of F-calprotectin (**c**) compared to other patients. Dot plot, median values marked by a horizontal line. *p < 0.05, **p < 0.005. EULAR European League Against Rheumatism

Patients with severe dysbiosis had higher disease activity, as evaluated by the ESSDAI, compared to the other pSS patients (13 (5; 16) vs 5 (1; 10); *p* = 0.049) (Fig. 2a). Similar findings were found for the ClinESSDAI (12 (4; 16) vs 5 (0; 9); *p* = 0.049). In line with these findings, hypocomplementemia was more pronounced amongst pSS patients with severe dysbiosis as reflected by lower serum levels of C4 (0.11 (0.07; 0.14) vs 0.17 (0.14; 0.21) g/L; *p* = 0.004) (Fig. 2b). pSS patients with severe dysbiosis had significantly higher F-calprotectin (110 (61; 330) vs 33 (20; 74) μg/g; *p* = 0.001) (Fig. 2c) compared to the other patients. The associations remained when excluding patients with milder dysbiosis and comparing patients with DIS 5 only to patients with DIS 1–2 (data not shown).

Neither disease duration, smoking, classical symptoms of pSS (as evaluated by the ESSPRI), IgG, IgM, or IgA levels, or serological features of pSS was associated with dysbiosis or severe dysbiosis in pSS patients. IBS-like symptoms were not more common in pSS patients with severe dysbiosis (Table 2).

**Table 2:** Clinical features in patients with primary Sjögren’s syndrome with and without severe dysbiosis

	Dysbiosis Index Score 1–4 (*N* = 33)	Dysbiosis Index Score 5 (*N* = 9)	*p* value
Age (years)	61 (50; 68)	62 (58; 71)	0.534
ESSDAI total score	5 (1; 10)	13 (5; 16)	0.049*
ClinESSDAI total score	5 (0; 9)	12 (4; 16)	0.049*
ESSPRI total score	6 (5; 8)	7 (6; 8)	0.224
IgG (g/L)	13.2 (10.0; 17.1)	12.4 (10.2; 22.0)	0.718
Complement component 3 (g/L)	1.01 (0.90; 1.21)	0.98 (0.79; 1.10)	0.608
Complement component 4 (g/L)	0.17 (0.14; 0.21)	0.11 (0.07; 0.14)	0.004**
Anti-SS-A antibody seropositives (%)	78	76	1.000
Anti-SS-A antibody seropositives (%)	45	56	0.714
Lip biopsy—focus score ≥ 1 (%)	85	86	1.000
Irritable bowel syndrome-like symptoms (%)	39	57	0.425
Fecal calprotectin (μg/g)	33 (20; 74)	110 (61; 330)	0.001**
Using glucocorticoids (%)	27	67	0.049*
Using anti-malarials (%)	24	44	0.406
Using proton pump inhibitors (%)	33	44	0.698
Using NSAIDs (%)	39	33	1.00

Comparison of clinical characteristics in primary Sjögren’s syndrome patients with severe dysbiosis (Dysbiosis Index Score 5) vs subjects with eubiosis or mild to moderate dysbiosis (Dysbiosis Index Score 1-4). Results presented as median (interquartile range) or fraction of patients with abnormal findings (%)

*ClinESSDAI* Clinical EULAR Sjögren’s Syndrome Disease Activity Index, *ESSDAI* EULAR Sjögren’s Syndrome Disease Activity Index, *ESSPRI* EULAR Sjögren’s Syndrome Patient Reported Index, *EULAR* European League Against Rheumatism, *NSAID* nonsteroidal anti-inflammatory drug

*p < 0.05

**p < 0.01

Usage of GCs was more common among patients with DIS 5 (67 vs 27%; *p* = 0.049). However, concomitant use of other medications (including the use of PPI, NSAIDs, and AMA) were not associated with severe or moderate dysbiosis in pSS patients (Table 2).

Subanalysis of the GA-map™ Dysbiosis Test made it possible to explore the abundance of 15 separate clades or species of bacteria. A significantly higher fraction of pSS patients exhibited decreased levels of bacteria from the genera *Bifidobacterium* (38 vs 3%; *p* < 0.001) and *Alistipes* (19 vs 3%; *p* = 0.017) in comparison to control subjects. There was also a tendency toward lower levels of *Faecalibacterium prausnitzii* among the pSS subjects, but this difference was not statistically significant (*p* = 0.061).

## Discussion

In the current study, we found that severe intestinal dysbiosis was a prevalent finding in pSS, affecting 21% of studied patients. In this cohort of consecutive pSS patients, severe intestinal dysbiosis was associated with both clinical and laboratory signs of systemic disease activity as well as with laboratory signs of GI tract inflammation, as evaluated by F-calprotectin.

Eubiosis and mild or moderate dysbiosis (DIS 1–4) were prevalent both in the pSS group and the control group. The relatively high fraction of intestinal dysbiosis in the control group differs from a previous report where only 16% of healthy adults exhibited DIS ≥ 3 [27]. However, the controls described previously were younger (mean age 41 years) [27] than the age-matched and sex-matched controls of the current study (mean age 60 years). Also, in our control group we had few exclusion criteria (ongoing GI or rheumatic disease), which is in contrast to the previous report. However, severe intestinal dysbiosis was significantly more common in pSS patients (21%) than in age-matched and sex-matched controls (3%), indicating an altered intestinal microbial balance in a subgroup of pSS patients.

Intestinal dysbiosis has been found to be a feature of several rheumatic diseases, including rheumatoid arthritis, systemic lupus erythematosus, systemic sclerosis, and pSS [10, 16, 20], as well as IBD [30]. Intestinal dysbiosis has hitherto been studied most extensively in IBD patients, in whom a decreased diversity in gut microbiota, increased numbers of bacteria driving inflammatory activity, as well as decreased numbers of bacteria with immunoregulatory effects have been demonstrated [30]. In rheumatic diseases as well as in IBD, it is still a matter of debate whether intestinal dysbiosis is a primary or secondary phenomenon of disease [31]. In animal models of IBD, loss of immunoregulatory bacteria and addition of disease-promoting bacteria contribute to disease activity, supporting a primary disease-driving role for intestinal dysbiosis in IBD [32]. Recently, two small studies reported oral [19] as well as intestinal [16] dysbiosis in pSS patients. In the prior study, the bacterial diversity was found to be lower in pSS patients, with normal salivary flow [19], thus implying that oral dysbiosis can occur irrespective of whether salivary flow was decreased or not. In the latter study, signs of dysbiosis were found both in the oral cavity and in stool, and intestinal dysbiosis was associated with disease severity [16].

In the current study, we found that clinical disease activity, as evaluated by the ESSDAI and the ClinESSDAI, as well as laboratory signs of disease activity were associated with severe intestinal dysbiosis. These findings are reminiscent of the findings in the study by de Paiva et al. [16] showing an inverse correlation between fecal microbial diversity and combined ocular and systemic disease activity in pSS patients. In our study, patients with severe dysbiosis more often required treatment with low-dose GCs, which we suggest reflects systemic disease activity, but we cannot exclude that GC treatment causes dysbiosis. Indeed, some animal studies have indicated a direct effect of GCs on the microbial balance in the intestine [33, 34]. Of interest, treatment with PPI, NSAIDs, or AMA was not associated with severe intestinal dysbiosis in our study, reminiscent of findings in previous studies [35, 36].

F-calprotectin, a marker of GI inflammation, was significantly increased in pSS patients with severe intestinal dysbiosis, which is similar to findings in patients with systemic sclerosis [21], and suggests that an imbalance in microbiota in the gut of pSS patients coincides with GI inflammation. However, a potential causality remains to be determined and it can only be speculated whether intestinal dysbiosis drives the inflammatory process in pSS or is merely a consequence of systemic disease affecting the GI tract [37]. One hypothesis, however, is that an imbalance in the gut microbiota in the genetically susceptible individual leads to an increase in T_H_17 cells in the gut [38], which in turn enter the circulation and migrate to the exocrine glands, and possibly other tissues, to drive inflammation and development of both exocrine disease and EGMs in pSS.

We report low levels of bacteria from the genera *Alistipes* and *Bifidobacterium* in pSS patients. Interestingly, low levels of *Alistipes* have been reported previously in psoriatic arthritis and Crohn’s disease [39, 40] and low levels of *Bifidobacterium* have been reported previously in both rheumatoid arthritis and Crohn’s disease [41, 42]. It remains to be explored why these specific alterations are found in clinically disparate diseases.

In contrast to patients with elevated F-calprotectin, patients with IBS-like symptoms did not display more severe dysbiosis than other patients. Instead, severe dysbiosis was as common among these subjects as in the control group. This finding is in contrast to previous reports in comparing IBS with healthy controls. The etiology of IBS and IBS-like symptoms is unknown, but is supposed to be multifactorial. Our findings may indicate that functional bowel symptoms associated with pSS have a different etiology compared to idiopathic IBS [9, 27].

To better understand the impact and importance of intestinal dysbiosis in pSS, future studies should investigate intestinal dysbiosis in larger samples of pSS patients. Ideally, we would like to study the intestinal microbiome longitudinally and in relation to factors likely to affect the intestinal microbiota (e.g., probiotics, prebiotics, antibiotics, specific diets, and anti-inflammatory medication). To further elucidate the important question of causality, further experimental animal studies in line with the previous publication by Paiva et al. [16] are warranted.

## Conclusions

Severe intestinal dysbiosis is prevalent in pSS and associated with both clinical and laboratory markers of disease activity as well as with laboratory signs of GI inflammation.

## Abbreviations

AECG: American–European Consensus Group
AMA: Anti-malarials
C3: Complement component 3
C4: Complement component 4
ClinESSDAI: Clinical EULAR Sjögren’s Syndrome Disease Activity Index
DIS: Dysbiosis Index Score
DMARD: Disease-modifying anti-rheumatic drug
EGM: Extraglandular manifestations
ESSDAI: EULAR Sjögren’s Syndrome Disease Activity Index
ESSPRI: EULAR Sjögren’s Syndrome Patient Reported Index
EULAR: European League Against Rheumatism
F-calprotectin: Fecal calprotectin
GC: Glucocorticoid
IBD: Inflammatory bowel disease
IBS: Irritable bowel syndrome
NSAID: Nonsteroidal anti-inflammatory drug
PPI: Proton pump inhibitors
pSS: Primary Sjögren’s syndrome
